# The relationship between interoception of breathing, anxiety, and resting-state functional connectivity in the brain

**DOI:** 10.3758/s13415-025-01328-7

**Published:** 2025-08-20

**Authors:** Isabella M. Chemis, Laura Köchli, Stephanie Marino, Bruce R. Russell, Klaas Enno Stephan, Olivia K. Harrison

**Affiliations:** 1 Department of Psychology, University of Otago, Dunedin, New Zealand; 2 Translational Neuromodeling Unit, Institute for Biomedical Engineering, University of Zurich and ETH Zurich, Zurich, Switzerland; 3 Department of Pharmacy, University of Otago, Dunedin, New Zealand; 4 Max Planck Institute for Metabolism Research, Cologne, Germany

**Keywords:** Interoception, Breathing, Anxiety, Functional connectivity

## Abstract

Impaired breathing-related interoceptive abilities have been associated with adverse outcomes, including higher levels of anxiety. However, brain connectivity patterns related to poor interoception, and how these may be modulated by anxiety, are poorly understood. This exploratory study investigated connectivity profiles associated with breathing-related interoceptive abilities in 65 volunteers who underwent ultrahigh-field (7 Tesla) "resting-state" magnetic resonance imaging (rs-fMRI), as well as completed a breathing-related interoceptive task and an anxiety questionnaire. The breathing task measured four aspects of interoceptive ability (sensitivity, decision bias, metacognitive bias, and insight), which served alongside anxiety to explain amygdala connectivity in the rs-fMRI data. We observed that connectivity between bilateral amygdala and insula cortex was linked to the level of confidence ascribed to respiratory-related interoceptive judgements (metacognitive bias), while left-lateralised connectivity between amygdala and insula cortex was associated with a worsened ability to detect inspiratory resistances (interoceptive sensitivity). Both reductions in confidence and sensitivity correlated weakly with heightened anxiety levels at a behavioural level. By contrast, the connectivity differences across levels of metacognitive bias and interoceptive sensitivity were not accounted for by anxiety. Our findings could suggest that, in the general population, connectivity between amygdala and insula cortex is linked to breathing-related interoceptive processes in a manner that is largely independent of anxiety.

**Supplementary Information:**

The online version contains supplementary material available at 10.3758/s13415-025-01328-7.

## Introduction

While anxiety disorders are among the most ubiquitous of all mental health conditions (World Health Organization, 2017), almost everyone will experience transient periods of anxiety (Tovote et al., 2015). In fact, subclinical yet elevated levels of anxiety have been reported to impact twice as many individuals as those with Generalised Anxiety Disorder (the most common anxiety disorder) and are associated with periods of significant distress and impairment (Haller et al., 2014). Furthermore, both the rates of anxiety disorders and anxiety within the general population have risen in response to the global COVID-19 pandemic (Saeed et al., 2022; Salari et al., 2020). Thus, understanding anxiety behaviours and identifying effective strategies for managing deleterious anxious symptomology is more important than ever before.

Interoception—the perception of internal body states (Khalsa et al., 2018)—is inextricably intertwined with allostatic regulation (Toussaint et al., 2024) and thought to be disrupted in relation to anxiety (Harrison et al., 2021c; Paulus, 2013; Paulus & Stein, 2010). As such, interoception has become a recent focus of psychopathology research (Brewer et al., 2021; Harrison et al., 2021c; Khalsa et al., 2018; Murphy et al., 2017; Nord et al., 2021). For example, alterations in interoceptive processes have been reported for anxiety (Harrison et al., 2021c; Tumati et al., 2021), depression (Chan et al., 2023; Nord et al., 2021; Smith et al., 2021), feeding and eating disorders (Kerr et al., 2016), fatigue (Rouault et al., 2018), and substance use disorders (SUD) (Smith et al., 2022). Owing to this widespread occurrence amongst clinical populations, atypical interoception has been proposed to operate as a general vulnerability factor to a range of neuropsychiatric conditions, disrupting effective interactions between the brain and the body (Murphy et al., 2018; Nord et al., 2021).

One interoceptive model proposed by Garfinkel & Critchley (2015) identifies different facets of interoceptive ability. Three key aspects described in their model include interoceptive sensitivity (accuracy in correctly identifying internal changes), interoceptive sensibility or metacognitive bias (subjective assessment of an individual’s ability to accurately perceive interoceptive signals), and interoceptive insight (the extent to which one’s own confidence in their interoceptive ability is congruent with their accuracy in detecting interoceptive signals) (Garfinkel et al., 2015). These interoceptive abilities are believed to uniquely alter the degree to which body signals can be either detected or (correctly) appraised (Harrison et al., 2021c). The current project considered all facets of interoceptive ability outlined in the Garfinkel & Critchley’s (2015) model as well as decision bias; another aspect of interoceptive ability that describes one’s tendency to report the presence or absence of an internal signal (Harrison et al., 2021c).

Respiratory changes are a common feature of an anxiety response (American Psychiatric Association, 2013), making the perception of breathing-related cues an important and amenable target for anxiety-related interoception research (Harrison et al., 2021c; Paulus, 2013). Furthermore, previous work has identified an association between reduced respiratory-related interoceptive abilities and heightened levels of anxiety, both within healthy and clinically anxious populations (Garfinkel et al., 2016b; Harrison et al., 2021c; Paulus, 2013; Tiller et al., 1987; von Leupoldt et al., 2011). Therefore, identification of the processes that facilitate these interoceptive deficits could have important implications for the understanding of anxiety mechanisms.

Finally, within the brain circuitry related to anxiety, the amygdala is believed to be a central constituent of anxiety processes (LeDoux et al., 1988). The amygdala is thought to coordinate both the autonomic physiological response and the higher-order cognitive reaction to danger (LeDoux et al., 1988; Liddell et al., 2005). The amygdala is also highly connected to the insula cortex (IC) (Höistad & Barbas, 2008; Stein et al., 2007), a key structure in the interoceptive processing pathway, integrating communication between the brain and body via multiple feedback loops (Berntson & Khalsa, 2021). These loops function to maintain homeostasis within the internal environment (Berntson & Khalsa, 2021; Toussaint et al., 2024) and have also been found to pervasively influence aspects of cognition, emotion, and behaviour (Critchley & Harrison, 2013).

Interestingly, very little research has utilised objective measures of interoceptive ability paired with neuroimaging techniques, particularly within the functional connectivity domain. Therefore, the neural underpinnings of these abilities remain largely ambiguous. Studies using task-based functional MRI techniques during heart beat counting/detection tasks have revealed that increased interoceptive accuracy was associated with increased IC, cingulate and opercular cortex activity (Critchley et al., 2004; Haruki & Ogawa, 2021; Pollatos et al., 2007). However, very little literature has directly investigated the relationship between brain connectivity, interoceptive abilities and anxiety and the existing literature on this topic is limited in its generalisability. For instance, rumination (defined as unconstructive negative thinking; a cognitive feature of depression and anxiety), was found to be linked to both alterations in interoception and brain connectivity (Li et al., 2022). However, the measure of interoception used was derived from a self-report scale and it is therefore unclear whether this relationship would be replicated with objective measures of interoceptive ability (Li et al., 2022). Therefore, the current exploratory study used existing data to investigate how amygdala connectivity at rest is altered in relation to both breathing-related interoceptive abilities and anxiety levels. It was hypothesised that the connectivity between the amygdala (as a central anxiety circuitry hub) and structures such as the IC (as a central interoceptive circuitry hub) may play an important role in the integration between anxiety and interoceptive (dys)function.

## Methods

The data were acquired at the Translational Neuromodeling Unit in the Institute of Biomedical Engineering at the University of Zurich and ETH Zurich, as part of a wider study (Harrison et al., 2021c). The study was approved by the Cantonal Ethics Committee Zurich (BASEC-No. 2017–02330), and all participants provided informed consent in line with the Declaration of Helsinki. The participants completed several tasks across two testing sessions (within 4 ± 3 days of each other), including questionnaires/interoceptive testing and a magnetic resonance imaging (MRI) session. The data relevant to this project that are described here include the results of an anxiety questionnaire, a breathing interoceptive task, and a task-free ("resting-state") functional MRI (rs-fMRI) session.

## Participants

Data were utilised from all available participants who completed the questionnaires, interoceptive task, and rs-fMRI session (*N* = 65) (Harrison et al., 2021c). A dataset of at least 54 participants would allow for 80% power within a regression analysis to detect a moderate effect size (*f*^*2*^ = 0.15) and a two-tailed alpha level of 0.05. All participants were right-handed, MRI-compatible, nonsmokers who had no history of severe mental or physical health conditions and provided written and informed consent to take part in the study. The mean age was 24.9 ± 4.5 (standard deviation [SD]) and included 32 females and 33 males. The Spielberger State-Trait Anxiety Inventory (STAI-T; score range 20–80) was used as a prescreening tool to ensure recruited participants spanned the spectrum of anxiety from low (trait anxiety scores between 20 and 25) to moderate levels of trait anxiety (scores > 35) (Spielberger et al., 1971).

## Procedures

### Questionnaires

During the first session, the participants completed a set of questionnaires, including the Generalised Anxiety Disorder Questionnaire (GAD-7) (Spitzer et al., 2006). While the GAD-7 was originally constructed to assess the extent of Generalised Anxiety Disorder in a clinical setting (Spitzer et al., 2006), it has also been validated as a measure of trait anxiety amongst the general population (Löwe et al., 2008). The GAD-7 was chosen as the main measure of anxiety for this project; notably, it was not used as a prescreening tool (unlike the STAI-T), allowing us to consider the whole group of participants across a spectrum of anxiety scores.

### Filter detection task

To measure aspects of breathing-related interoceptive ability, participants completed the Filter Detection Task (Harrison et al., 2021a). This task measures four aspects of interoceptive ability related to the detection of an inspiratory resistance; interoceptive sensitivity (operationalised by the minimum magnitude of inspiratory resistance that the participant was able to accurately detect); decision bias (the tendency to indicate the presence or absence of a resistance); metacognitive bias (the average level of confidence ascribed to decisions regarding the presence/absence of an inspiratory resistance); and metacognitive insight (the congruency between confidence and performance on the task).

To perform the FDT, a yes/no decision paradigm was employed. Participants had to decide on each trial whether an additional inspiratory resistance was applied to the system. To achieve this, participants breathed through a custom-built breathing circuit (Harrison et al., 2021a), where a single-use filter mouthpiece (Powerbreathe International Ltd., Warwickshire, UK—Product SKU PBF03) was attached to a one-way non-rebreathing t-piece valve (Hans Rudolf, Kansas City, MO—Product 1410/112,622) and a 22 mm diameter, 2 m length inspiratory breathing tube (Intersurgical Ltd.—Product 1,573,000) and two additional baseline filters (1 × Intersurgical Ltd.—Product 1,541,000, and 1 × GVS, Lancashire, UK—Product 4222/03BAUA). Connected to the end of the inspiratory circuit (placed behind the participant’s back, out of view) was an empty sham filter (GVS Filter Technology—Product 2800/22BAUF, with the filter membrane removed). Full details are provided in the supplementary material.

In each trial, participants first took three baseline breaths, after which either a “resistance” or “sham” condition was applied for a further three breaths. In the sham condition, the empty filter was removed from the circuit and then replaced to replicate any movement or noise associated with the resistance condition. In the resistance condition, the empty sham filter was replaced with a stacked set of breathing filters (GVS Filter Technology—Product 2800/22BAUF) to add a specific amount of inspiratory resistance to the circuit. Each filter provides a resistance < 0.48 cm H_2_O/L.sec-^1^, and the number of filters applied was determined by a custom staircase algorithm described in Harrison et al. (2021a). The algorithm aims to hold task performance ideally between 65–80%, utilising the results of the preceding trials to estimate the underlying accuracy of the performer at the current level of resistance. As such, the difference in beta cumulative distribution functions for 80% and 65% is calculated at each trial, in a similar vein to the QUEST algorithm (Watson & Pelli, 1983). If the probability that the underlying accuracy of the performer is within this range falls below a prespecified 20% threshold, the algorithm suggests the addition of a filter (if underlying performance is below 65%) or the removal of a filter (if the underlying performance is above 80%) to decrease or increase task difficulty, respectively. Owing to the limited resolution of step sizes (i.e. level of resistance) available, the limited number of possible trials due to participant fatigue, and the variability of breathing physiology from trial-to-trial, this simple algorithm is robust, does not rely on prior assumptions as to the shape of the psychometric function, and does not run the risk of non-convergence that can be observed with more complicated algorithms such as QUEST. Full details and validation of the algorithm performance can be found in Harrison et al. (2021a).

Upon the completion of each trial (6 breaths total), participants were asked to indicate whether they believed a resistance had been applied to their breathing (yes/no) and how confident they were in that decision (on a numerical rating scale from 1–10; 1 being not at all confident and 10 being extremely confident). The task was finished once 60 trials at one filter level were complete, although if the level of resistance changed and then returned to the original level, the trial count would continue on from the previous trials at the same resistance. The mean ± std for the total number of trials completed (to achieve the 60 trials included in further analyses) was 73 ± 14 trials. The resistance and sham trials were presented in a pseudo-randomised order, with an equal number of each type shuffled over blocks of ten trials. A visual overview of the trial and task structure is available in the supplementary material. All task and analysis code is publicly available and free to download: https://github.com/translationalneuromodeling/tapas/tree/master/task/FDT.

### Resting-state functional magnetic resonance imaging

**Fig. 1:**
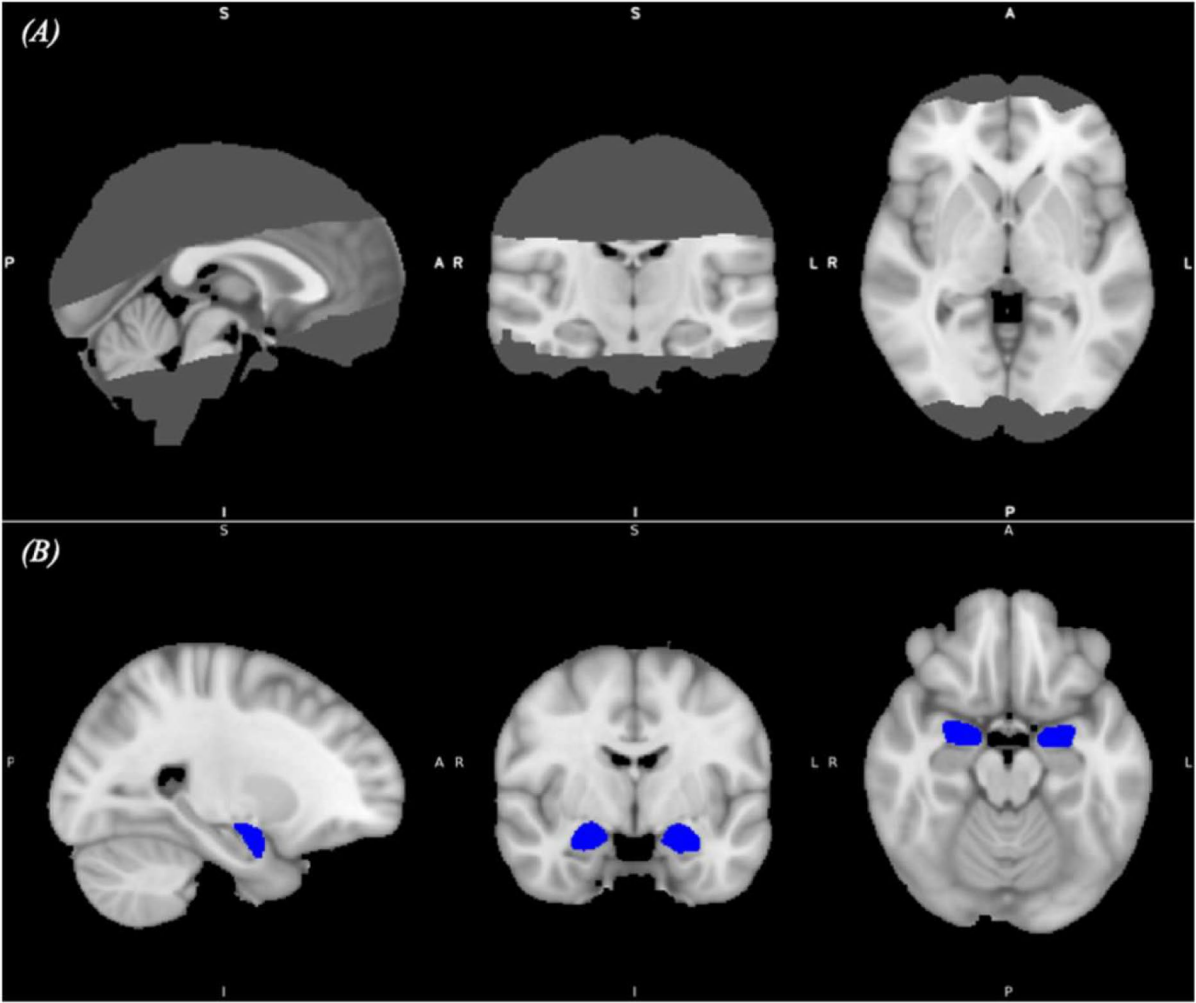
**(A)** The reduced field of view imaged during resting-state fMRI. The final mask is a minimum map across all participants once warped into standard MNI space.** (B) **Bilateral amygdala masks (shown in blue) from the Harvard–Oxford Subcortical Atlas overlaid on a T1 template in standard space (MNI-152). These masks defined the regions of interest for extracting the functional time series for each participant

MRI measurements took place in the second study session. This study utilised the data of a rs-fMRI scan at high field strength, obtained using a 7 Tesla scanner (Philips Medical Systems: Achieva, Philips Healthcare, Amsterdam, the Netherlands) with a 32-channel Head Coil (Nova Medical, Wilmington, MA). Scans (T2*-weighted, gradient echo EPI) were obtained using a reduced field of view (FOV; Fig. 1A), with an axial-oblique volume centred over the amygdala, IC, anterior cingulate cortex (ACC), regions of the prefrontal cortex (PFC), and midbrain. The FOV comprised 32 slices (sequence parameters: TE 30 ms; TR 2.3 s; flip angle 75°; voxel size 1.5 × 1.5 × 1.5 mm; slice gap 0.15 mm; SENSE factor 3; ascending slice acquisition), with 250 volumes (scan duration 9 min 35 s). Additionally, both whole-brain EPI scans (96 slices) and T1-weighted structural scans (200 slices; MPRAGE, sequence parameters: TE 4.6 ms; TR 10 ms; segment-TR 3,000 ms; TI 1,000 ms; flip angle 8°; voxel size 0.8 × 0.8 × 0.8 mm; bandwidth; 153.1 Hz/Px; sagittal slice orientation) were obtained. During the rs-fMRI scan, participants were asked to keep their eyes open and fixate on a white cross on a black background on the screen.

## Data analysis

### FDT analysis

The four outcome measures of interoceptive ability (interoceptive sensitivity, decision bias, metacognitive bias and metacognitive insight) were obtained through the FDT. First, interoceptive sensitivity was quantified by the number of filters at which an inspiratory resistance can be consciously perceived by the participant at an above-chance level (60–85% accuracy). As such, low numbers of resistance filters are representative of increased interoceptive sensitivity to inspiratory resistance, as this indicates a lower load is required for the participant to reliably perceive it.

Second, decision bias (the tendency to report the presence/absence of a resistance) was computed for each participant using the HMeta-d toolbox (Fleming, 2017). The HMeta-d model utilises concepts of Signal Detection Theory (SDT) (Stanislaw & Todorov, 1999) to both separate and quantify signal sensitivity (d’) and decision bias (c) (both of which influence task performance). Positive values of the decision bias measure (c > 0) indicate a tendency to underreport the presence of a resistance on FDT trials, while negative values (c < 0) are indicative of the tendency to over-report the presence of a resistance.

Third, the metacognitive bias measure denotes one’s tendency to describe their perceptual decisions on the FDT with high or low levels of confidence. It is therefore calculated as the average confidence score across trials; higher levels of metacognitive bias represent a greater propensity to ascribe high confidence ratings to decisions on the FDT.

Finally, metacognitive insight (congruency between decision confidence and performance) was also calculated using the HMeta-d model. This first uses concepts of SDT to calculate “absolute metacognitive insight” known as meta-d’ (Fleming, 2017; Maniscalco & Lau, 2012). The meta-d’ value for each participant is then divided by their d’ value, resulting in an estimate of metacognitive insight known as “Mratio.” Although the number of perceptual filters at perceptual threshold (60–85% accuracy) operates as the measure of interoceptive sensitivity in the FDT, d’ captures any residual differences in accuracy (between 60–85%). The Mratio thus quantifies metacognitive insight whilst controlling for any residual differences in underlying task performance between individuals. Given the low number of trials in interoceptive experiments, the parameters of the HMeta-d model are estimated using a hierarchical Bayesian scheme. The data from all participants are therefore used to inform and constrain the estimates of the single-subject meta-d’ values, which helps to improve the robustness of the single-subject measures of metacognitive insight (Harrison et al., 2021a). A plot of the posterior distribution and mixing of sample chains from the sampler (JAGS), as well as a qualitative comparison of the observed and model estimates are provided as evidence of model convergence in the supplementary material. Additionally, the adequacy of the HMeta-d model to accurately recover Mratio values over 60 trials is demonstrated via simulation analyses presented in the supplementary material and in Harrison et al. (2021a). The HMeta-d model was implemented by using a MATLAB toolbox, available at https://github.com/smfleming/HMeta-d (Fleming, 2017).

To assess collinearity between FDT measures, a correlation matrix was calculated between each variable pair. Correlations were considered significant at *p* < 0.05, uncorrected for multiple comparisons and clearly marked as exploratory. Furthermore, in accordance with reviewer suggestions, we tested the specificity of the metacognitive bias findings by correlating these scores with additional available questionnaire results from the Multidimensional Assessment of Interoceptive Awareness; MAIA (Mehling et al., 2012) as an additional measure of subjective metacognitive bias (i.e., interoceptive sensibility (Garfinkel et al., 2015)). Additionally, we tested the generalisability of the metacognitive bias findings by correlating these scores with more general measures of affect, utilising the Positive and Negative Affect Schedule scores (both positive and negative scores) (Watson et al., 1988).

### Resting-state fMRI analysis

The resting-state data pre-processing and analysis were performed using FSL version 6 (the Oxford Centre for Functional Magnetic Resonance Imaging of the Brain Software Library, Oxford, UK) (Jenkinson et al., 2012). Standard MRI pre-processing of the raw data was conducted, including brain extraction using BET to remove non-brain structures from the T1 weighted structural images (Smith, 2002), and motion correction of the functional scans using MCFLIRT (Jenkinson & Smith, 2001). Data de-noising was executed using independent component analysis with manual classification of noise components, using automatic estimation for the number of components for each participant (Griffanti et al., 2017). The images from each participant’s resting-state scan were registered to their T1-weighted structural scan using the BBR cost function in FLIRT (6DOF) (Jenkinson et al., 2002). All images were registered to 1 mm standard space (MNI152), by using a combination of FLIRT and FNIRT to perform affine (12DOF) and nonlinear (12 + DOF) registration, respectively (Andersson et al., 2007).

The resting-state data were analysed by using a seed-based correlation analysis (SCA), with the left and right amygdala serving as the primary seeds of interest. Amygdala masks were generated using the Harvard–Oxford Subcortical Atlas (Desikan et al., 2006) (Figure 1B). These masks were thresholded at an amygdala atlas structure probability of 90% and binarised before being transformed from standard to functional space for each participant (and re-thresholded at a probability of 90% within individual-subject space). From here, mean time series within these masks were extracted from each subject’s left and right amygdala separately to minimise assumptions and fully explore the amygdala connectivity profiles within this pilot study. These time series data were then used as regressors (alongside corresponding temporal derivatives) in general linear models (GLMs), separately for the left and right amygdala using FEAT, to identify voxels throughout the participant’s brain that showed correlated activity with the seed region of interest. The product of these analyses were hemisphere-specific individual seed-based functional connectivity (FC) maps for each participant.

A middle-level fixed effects analysis was then performed to calculate the average and any differences in amygdala connectivity between the two hemispheres of the brain. These middle-level FC maps were then used in higher-level mixed-effects analyses to elucidate the relationship between the FC of the amygdala seeds and each aspect of interoceptive ability and trait anxiety level. Specifically, each FDT measure served as an explanatory variable (EV) in two higher-level GLMs—one that included GAD-7 (to control for potential confounding effects of anxiety) and one that did not. A separate GLM was additionally used to consider the amygdala connectivity related to GAD-7 scores alone. In each GLM, the EVs (interoceptive and anxiety regressors) were de-meaned and separated into scores for males and females separately to account for any differences in gender (Lewinsohn et al., 1998). This approach was chosen such that we could selectively consider the moderating effect of anxiety on each interoceptive dimension separately. On request from a reviewer, we conducted a sensitivity analysis comparing amygdala connectivity when metacognitive bias served as the primary EV to the connectivity observed when each of the MAIA and PANAS (positive; PANAS-P) scores were utilised as the EV. Finally, an additional set of matching exploratory analyses were conducted using the anterior insula (aIC) as a seed region. The aIC region of interest was defined by using a mask taken from the Brainnetome Atlas of the bilateral ventral and dorsal aIC (Fan et al., 2016), thresholded at an aIC atlas structure probability of 50% (owing to the relatively larger size of this mask compared with the amygdala). Permutation testing for each contrast was conducted via the randomise tool in FSL (Winkler et al., 2014), with statistical significance declared at *p* < 0.05 using threshold-free cluster enhancement, with family-wise error correction for multiple comparisons (Smith & Nichols, 2009).

## Results

### Behavioural data summary

**Fig. 2:**
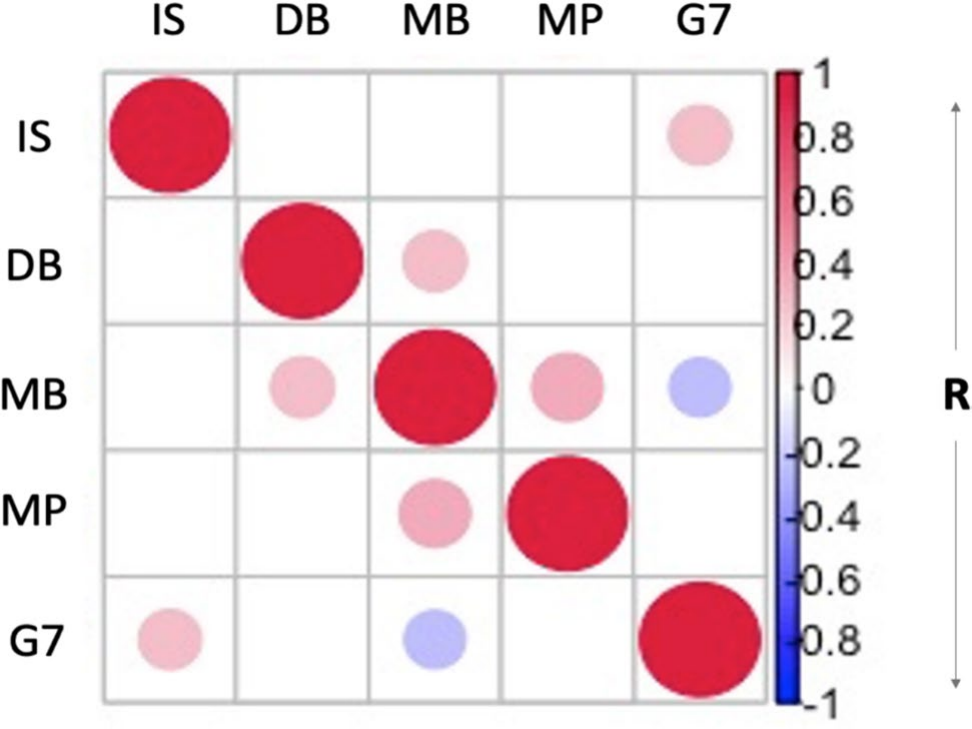
Matrix depicting the pairwise correlation between the behavioural variables: interoceptive sensitivity (IS), decision bias (DB), metacognitive bias (MB), metacognitive insight (MP) and GAD-7 scores (G7). The circles reflect the presence of a significant (*p* < 0.05) correlation (Pearson’s R) between the variable pair, with the colour and size demonstrating the directionality and strength of the relationship. The matrix is symmetric above and below the diagonal. Interoceptive sensitivity is an inverse measure (whereby high values are indicative of low interoceptive sensitivity); thus the positive correlation depicted between this measure and GAD-7 is indicative of a negative correlation between these two behavioural outcomes

Across all 65 participants, the average GAD-7 score was 2.85 (± 2.49) (mean ± standard deviation), with a range of 1–10. For the FDT, the average filter load applied for performance on the task to be maintained at 60–85% accuracy was 3.69 (± 1.84) resistance filters (with increased filter load representing a decrease in interoceptive sensitivity). The average decision bias score c across the cohort was 0.09 (± 0.33) (with c > 0 depicting a tendency to report no resistance). Concerning the metacognitive measures, the average metacognitive bias (confidence from 1–10) score across participants was 6.22 (± 1.37), while the average Mratio (metacognitive insight score) was 0.84 (± 0.05). Correlation coefficients (Pearson’s R) between each two-variable pair that passed *p* < 0.05 (uncorrected for multiple comparisons) threshold are presented as exploratory findings in the correlation matrix below (Fig. 2; Supplementary Tables 1 and 2). Notably, this correlation analysis revealed GAD-7 was negatively correlated with metacognitive bias (R = − 0.26; *p* = 0.04) and positively correlated with the interoceptive sensitivity measure (R = 0.27; *p* = 0.03), although these did not survive FDR correction for multiple comparisons (Supplementary Table 3). Finally, we observed a significant correlation between metacognitive bias scores and an additional measure of metacognitive bias (MAIA scores) (R = 0.3; *p* = 0.02), as well as a significant correlation with more general positive affect (PANAS-P) (R = 0.37; *p* < 0.01) but not negative affect (PANAS-N) (R = − 0.22; *p* = 0.09).

### Resting-state data

The amygdala-seed resting-state FC (rsFC) analyses yielded significant connectivity differences between the amygdala and widespread cortical areas for both the metacognitive bias and interoceptive sensitivity measures (described below). These findings were denoted by clusters of voxels that had a time series significantly related to the amygdala seed time series, utilising threshold free cluster enhancement and *p* < 0.05 (family-wise error corrected for multiple comparisons). By contrast, the amygdala-seed analyses which utilised the GAD-7 and metacognitive insight measures alone as explanatory variables, returned no significant results. Finally, the analyses that employed decision bias as the sole explanatory variable returned no main effects, with minor gender interaction effects reported in the supplementary materials. Results from the exploratory aIC seed analyses are reported in the supplement.

### Metacognitive bias

#### Bilateral amygdala seeds

**Fig. 3:**
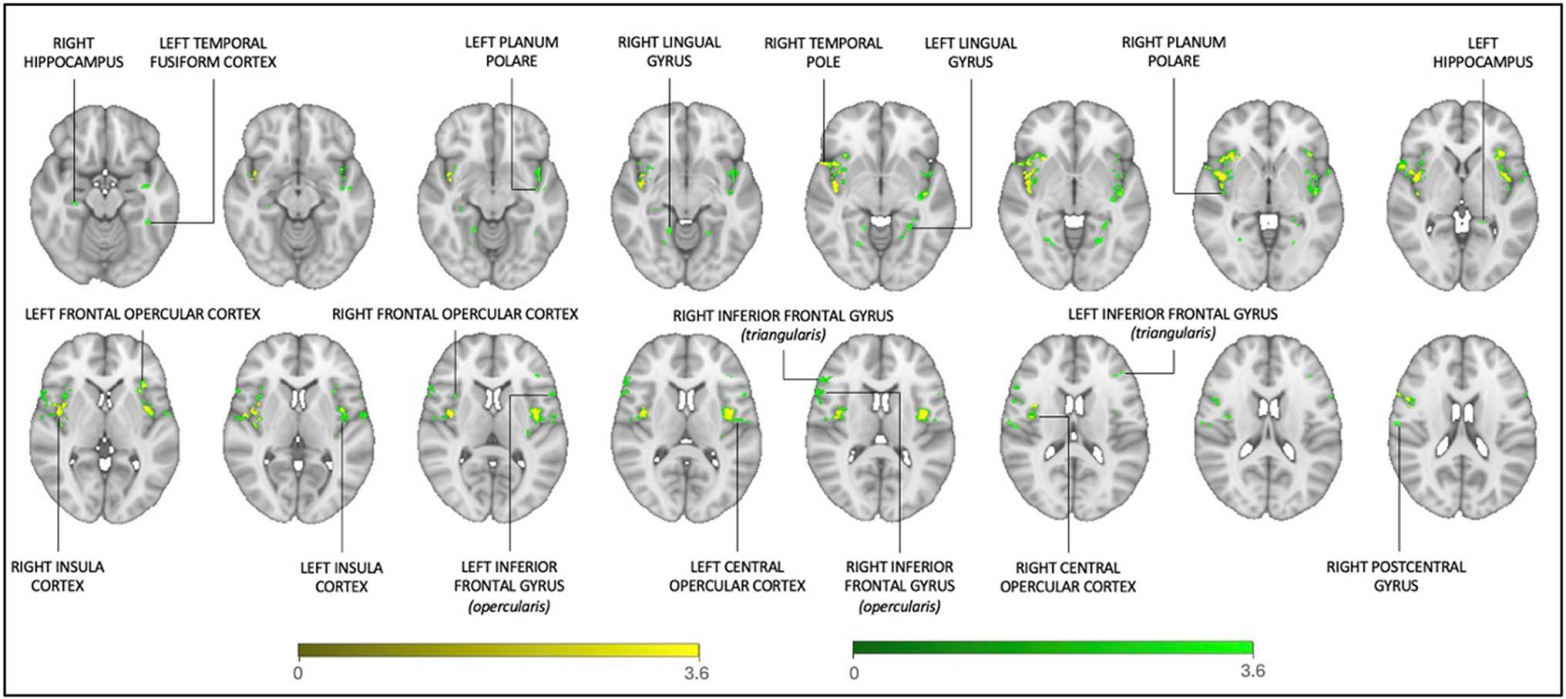
Regions demonstrating significant rsFC with the amygdala seed that correlated with metacognitive bias. The green clusters depict the Z-scores in regions that showed significant connectivity with the amygdala seed that correlated with metacognitive bias, while the yellow clusters (overlayed) illustrate the significant Z-scores for the connectivity clusters that remained after controlling for GAD-7. Statistical maps were derived using permutation testing (*p* < 0.05) and threshold-free cluster enhancement (family-wise error corrected for multiple comparisons). These maps were colour-rendered and superimposed on an MNI standard (1 × 1 × 1 × 1 mm) brain

Metacognitive bias was positively associated with rsFC between the bilateral amygdala seeds and a range of other brain regions: most prominently with the bilateral IC (disperse connections to anterior and middle insula; aIC and mIC), but also the bilateral central opercular cortices (CO), planum polare, lingual gyri, inferior frontal gyri, hippocampi, and the frontal opercular cortices, as well as the right temporal pole, precentral gyrus and postcentral gyrus, and the left temporal fusiform cortex (Fig. 3). After controlling for trait levels of anxiety (GAD-7 scores), the rsFC clusters in the lingual gyri, hippocampi, the right postcentral gyrus and the left temporal fusiform cortex were no longer significant. However, connectivity remained evident between the amygdalae seeds and the bilateral aIC and mIC, CO and planum polare, as well as the right inferior frontal gyrus (pars opercularis), temporal pole and precentral gyrus and the left frontal opercular cortex (Fig. 3). Additionally, our sensitivity analyses yielded no significant results, indicating that neither the PANAS-P nor MAIA scores served to explain the amygdala connectivity patterns related to metacognitive bias scores. However, some similarity was evident in the amygdala-insula connectivity for the MAIA x right amygdala contrast only – see supplementary material for details.

### Interoceptive sensitivity

#### Left amygdala > Right amygdala

**Fig. 4:**
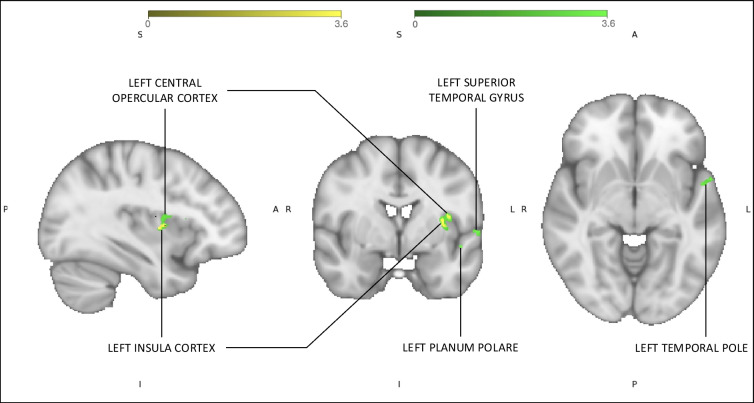
Regions exhibiting left > right amygdala rsFC correlated with decreased interoceptive sensitivity before and after controlling for GAD-7 scores. The green clusters depict significant Z-scores reflecting connectivity that correlated with decreased interoceptive sensitivity in the left > right amygdala seed before controlling for GAD-7 scores, while the yellow clusters (overlayed) illustrate the significant Z-scores for the connectivity clusters that remained after controlling for GAD-7. Statistical maps were derived using permutation testing (*p* < 0.05) and threshold-free cluster enhancement (family-wise error corrected for multiple comparisons). These maps were colour-rendered and superimposed on an MNI standard (1 × 1 × 1 × 1 mm) brain

In several brain regions, the degree of rsFC asymmetry between left versus right amygdala was found to be correlated with interoceptive sensitivity. Specifically, in the left aIC and mIC, CO, planum polare, superior temporal gyrus (anterior division), and the temporal pole, the difference of rsFC with left versus right amygdala was found to be correlated with worsened interoceptive sensitivity (Fig. 4). No significant differences were found for right amygdala > left amygdala related to any interoceptive measures. Finally, the difference in rsFC to the left mIC and CO with interoceptive sensitivity remained after controlling for GAD-7 (Fig. 4), while all other significant regions of connectivity did not endure after controlling for GAD-7 scores.

## Discussion

### Main findings

The current exploratory study investigated associations between amygdala rsFC and breathing-related interoceptive abilities—a topic that, to the best of our knowledge, has not been explored in prior literature. The results revealed that connectivity between the bilateral amygdala and aIC and mIC positively correlated with confidence in interoceptive decisions (metacognitive bias), while differences in FC between the aIC and mIC and left versus right amygdala was associated with worsened interoceptive sensitivity. In contrast, amygdala rsFC did not differ across levels of decision bias nor metacognitive insight. Behaviourally, anxiety scores displayed a weak negative correlation with both interoceptive sensitivity and confidence. Additionally, despite demonstrating no main effect on connectivity, anxiety scores controlled for some of the connectivity variance across the levels of metacognitive bias and interoceptive sensitivity.

### Metacognitive bias and amygdala rsFC

Most prominently, our analyses revealed that as amygdala-aIC/mIC connectivity increased so did the tendency to ascribe high levels of confidence to breathing-related perceptual decisions. Commonly deemed the hub of interoception, the IC is believed to be fundamental in generating an internal representation of one’s bodily state (Centanni et al., 2021; Simmons et al., 2013), while the amygdala is thought to play a central role in denoting salience to important stimuli in the internal and external environments (Davis & Whalen, 2001). It has therefore been posited that communication between the amygdala and the IC might be important for signalling the saliency of interoceptive signals when forming representations of bodily states (Baur et al., 2013; Paulus & Stein, 2010). Specifically, Baur et al. (2013) postulated that increases in amygdala-aIC connectivity could elevate the propensity for interoceptive signals to be conveyed as highly significant. This framework could in part explain the positive correlation between metacognitive bias (interoceptive confidence) and amygdala-aIC/mIC connectivity outlined by the present study; if an individual experiences respiratory-related interoceptive signals as more salient (related to augmented amygdala-IC salience signalling), they may experience more confidence relative to their interoceptive judgements. Importantly, this does not necessarily mean that enhanced amygdala-IC connectivity makes interoceptive perceptions more accurate but might instead make the IC’s portrayal of these signals more prominent (regardless of their correctness).

Furthermore, the rsFC clusters of significance between the amygdala and aIC/mIC (despite reducing in size) remained mostly unchanged after controlling for anxiety. We therefore postulate that within a healthy population, amygdala-IC connectivity related to interoceptive confidence is not strongly associated with anxiety, and instead is more purely linked to confidence in interoceptive percepts. Importantly, we also observed that the metacognitive bias scores were related to additional (theoretically similar) interoceptive questionnaire measures, such as the Multidimensional Assessment of Interoceptive Awareness, or MAIA. However, metacognitive bias scores were also related to more general levels of positive affect (but not negative affect). Therefore, while the increased connectivity between amygdala-aIC/mIC may also be reflective of more generalised levels of positive affect related to greater metacognitive bias rather than specific to interoceptive measures, we did not observe this when we compared these scores to amygdala rsFC.

### Reduced interoceptive sensitivity and asymmetric amygdala rsFC

The amygdala exhibited a lateralised connectivity profile associated with interoceptive sensitivity. Specifically, as perceptual sensitivity to a breathing resistance was reduced, the left amygdala became more strongly connected (compared to the right amygdala) to left-lateralised cortical regions (the left aIC/mIC, CO, planum polare, superior temporal gyrus, and the temporal pole) (Fig. 4). Additionally, after controlling for trait levels of anxiety, only the connectivity clusters in the left mIC and CO (often considered to constitute a functionally coupled unit Critchley et al., 2004; Pollatos et al., 2016)) remained significant. Thus, in contrast to all other significant regions, the connectivity differences between the amygdala and mIC/CO across interoceptive sensitivity levels may not be solely a result of trait anxiety differences.

The amygdala has previously been found to show lateralised activity in response to threatening stimuli. Specifically, Morris et al. (1999) found fearful facial expressions presented below conscious awareness to be preferentially processed by the right amygdala, while the left amygdala responded to consciously perceived threatening stimuli. They posited that this finding reflected a right-lateralisation of the subcortical threat response, whereby threatful stimuli are relayed quickly to subcortical brain regions to initiate reflexive reactions (Morris et al., 1999). As respiratory changes can be indicative of threat and involve the amygdala (Ziemann et al., 2009), it is possible that this lateralisation could be present in the conscious detection of inspiratory resistances. Specifically, a left-lateralised pattern of amygdala rsFC was present for those with reduced interoceptive sensitivity, potentially indicating a reliance on conscious breathing perceptions within interoceptive signal processing and cortical representation of body state.

Finally, the left amygdala (compared with the right amygdala) is believed to have a predominant role in negative emotions, such as fear and anxiety (Baas et al., 2004; Costafreda et al., 2008). In line with this notion, left-dominant amygdala-aIC connectivity has been observed in individuals with higher levels of anxiety (Baur et al., 2013; Jung et al., 2018)—a population known to catastrophise about body signals (such as respiratory changes) (Paulus, 2013) and show reduced interoceptive sensitivity to an inspiratory resistance (Harrison et al., 2021c; Tiller et al., 1987). Therefore, it is possible that individuals with increased left amygdala-IC connectivity might demonstrate an inflated anxiety response to the anticipation or perception of an inspiratory resistance and as a result less accurate perceptions of inspiratory resistance.

## Limitations and future directions

The current study employed a yes/no decision task for detecting inspiratory resistances. Utilising SDT with a yes/no task relies on assumptions of normality and equal variance (Stanislaw & Todorov, 1999), while two-interval forced choice (2IFC) configurations do not (Stanislaw & Todorov, 1999). Because we found no significant correlations between decision bias (which is only interpretable from a yes/no task) and trait anxiety nor rsFC, future investigations may choose to utilise 2IFC paradigms to minimise this effect (Nikolova et al., 2022). Additionally, this could be paired with a whole-brain FOV for neuroimaging, allowing for investigation of the neural correlates of respiratory-related interoceptive abilities at a network level across the whole brain. Furthermore, because this pilot study is exploratory in nature, the results should be interpreted with caution. Specifically, whilst multiple comparison correction was conducted within each analysis according to best practice procedures for fMRI, the interoceptive measures were analysed separately due to collinearity and the limited power of the study sample to overcome this issue. This limited power was evidenced by the lack of any significant results when each of the measures were combined into one analysis. Therefore, the risk of spurious results from the individual analyses remains a notable possibility. Thus, subsequent research is required with larger sample sizes to validate these findings. Finally, future studies might also focus on the relationship between interoception and clinically relevant levels of anxiety, and how this might be altered with either anxiety-centred (e.g., psychotherapy) or interoception-centred (e.g., floatation-rest, mindfulness meditation or exercise (Amaya et al., 2021; Feinstein et al., 2018)) treatment strategies.

## Conclusions

We found that rsFC between the amygdala and the IC is related to aspects of interoceptive ability within the breathing domain. Specifically, we identified bilateral increases in amygdala-aIC/mIC connectivity that correlated with heightened confidence in breathing-related interoceptive judgements, while left-lateralisation of amygdala-aIC/mIC rsFC was linked to a reduced ability to detect an inspiratory resistance. In addition, we found that levels of anxiety only minimally explained these amygdala-IC connectivity differences in our volunteers from the general population. Thus, our findings suggest that anxiety and breathing-related interoceptive abilities might not strongly share common neural pathways within individuals from the general population, despite their weak behavioural associations. However, the interaction between these two systems may change at clinical levels of anxiety (Bach, 2022; Grillon et al., 2019), which was not directly investigated here.

## Supplementary Information

Below is the link to the electronic supplementary material.

Supplementary file1 (PDF 4144 KB)

## Data Availability

Data sharing inquiries should contact tnu-datasharing@biomed.ee.ethz.ch.

## Code availability

Please contact the corresponding author for access to the code utilised in these analyses.

## Declarations

### Open practices statement

Data or materials for the experiments are available upon request and none of the experiments was preregistered.
