# Supplementary material for: The relationship between interoception of breathing, anxiety, and resting-state functional connectivity in the brain
Source: Cogn Affect Behav Neurosci. 2025 Aug 20;25(6):1795–806. doi: 10.3758/s13415-025-01328-7 (PMC12615565; doi:10.3758/s13415-025-01328-7)
Supplement: Supplementary file 1 — Supplementary file1 (PDF 4144 KB) [file 13415_2025_1328_MOESM1_ESM.pdf]

Supplementary Material:

The relationship between interoception of breathing, anxiety and resting-state functional connectivity in the brain

Isabella M. Chemis[1], Laura Köchli[2], Stephanie Marino[2], Bruce Russell[3], Klaas Enno Stephan[2,4], Olivia K. Harrison[1,2]

[1] Department of Psychology, University of Otago, Dunedin, New Zealand
[2] Translational Neuromodeling Unit, Institute for Biomedical Engineering, University of Zurich and ETH Zurich, Zurich, Switzerland
[3] Department of Pharmacy, University of Otago, Dunedin, New Zealand
[4] Max Planck Institute for Metabolism Research, Cologne, Germany

Corresponding authors:

Isabella M. Chemis

Email: isy.chemis@gmail.com

Department of Psychology

University of Otago

Olivia K. Harrison (née Faull)

Email: olivia.harrison@otago.ac.nz

Department of Psychology

University of Otago

1. Filter Detection Task Method and Data Modelling

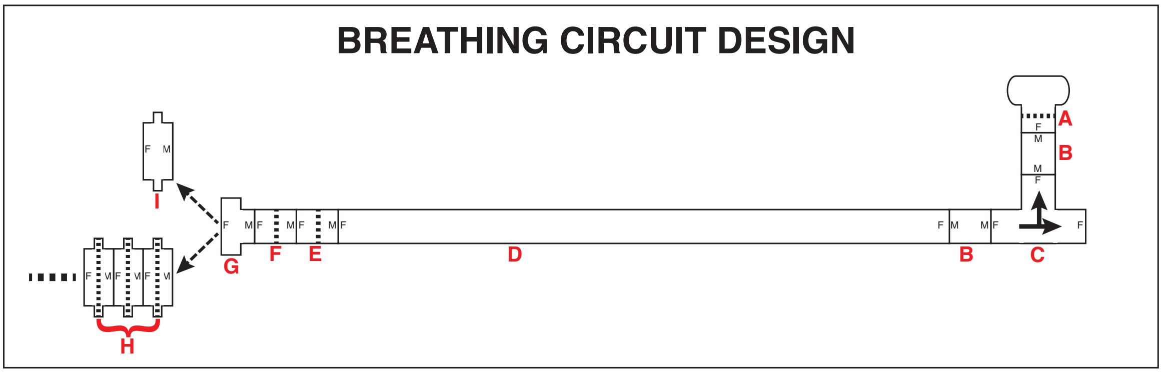

Supplementary Figure 1. Diagram of circuitry for the filter detection task. A single-use, bacterial and viral mouthpiece. A: Powerbreathe International Ltd., Warwickshire, UK - Product SKU PBF03) is attached to a 22 mm diameter connector. B: Intersurgical Ltd., Berkshire, UK - Product 1960000) and a t-shaped inspiratory valve. C: Hans Rudolf, Kansas City, MO, USA - Product 1410/ 112622), connected to a 2 m length of 22 mm diameter flexible tubing. D: Intersurgical Ltd. - Product 1573000) and two additional baseline filters (E: Intersurgical Ltd. - Product 1541000, and F: GVS, Lancashire, UK - Product 4222/ 03BAUA). A 22-30 mm. G: Intersugical Ltd. - Product 197100) adapter then allows the attachment of either a series of connected spirometry filters. H: GVS - Product 2800/17BAUF, Pressure at 30 L/min < 0.3 cm H2O, Resistance < 0.48 cm H2O/L.sec-1) or a sham filter – a spirometry filter shell with the inner bacterial protection pad removed (I). Figure adapted from Harrison et al. (2021a) under a CCBY licence.

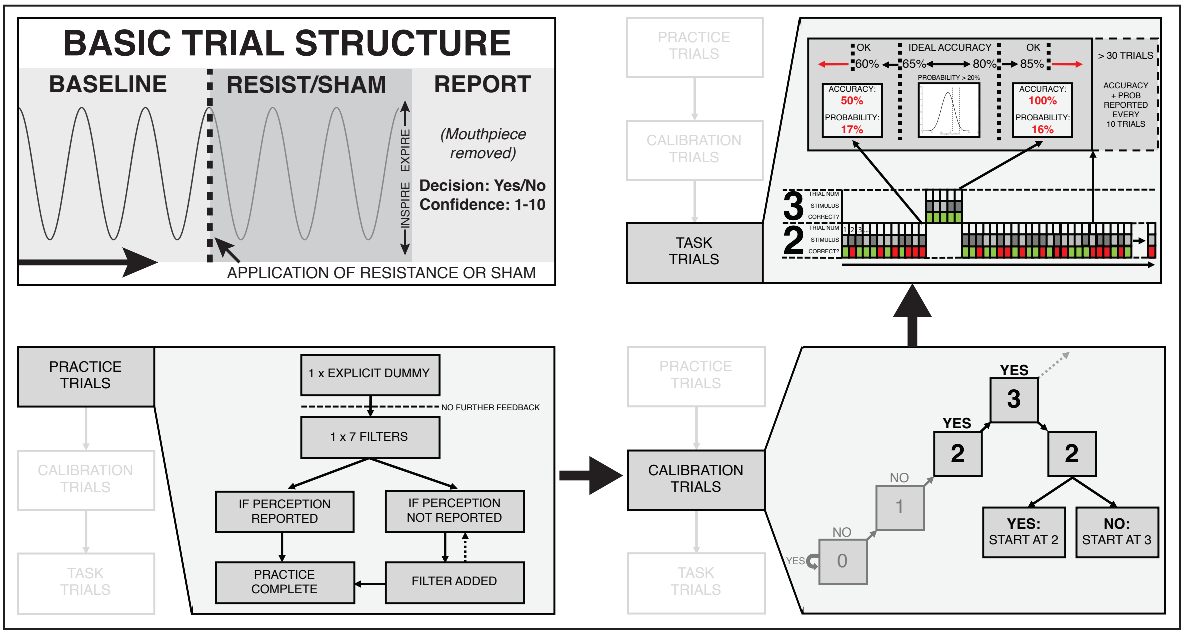

Supplementary Figure 2. Visualization of the task structure and performance algorithm. TOP LEFT: Overview of the basic trial structure for the task. Participants take three normal size/pace breaths (with the sham filter attached), and during the third exhalation (indicated by the participant raising their hand and the dotted line) the experimenter either swaps the sham for a number of stacked filters (to provide a very small inspiratory resistance) or removes and

replaces the sham filter. Following three more breaths, the participant removes the mouthpiece and reports whether they thought it a resistance was added ('Yes') or not ('No'), and how confident they are in their decision on any scale (here 1-10 used, with 1 = guessing and 10 = maximally confident in their decision). BOTTOM LEFT: Practice trials, consisting of an explicit dummy (where the participants are told a sham resistance is added), followed by a large load (7 filters) where no feedback is given (this is then maintained for the rest of the experiment). If no resistance is perceived in the Yes/No format or the answer is incorrect in the 2IFC format, filters are added until a correct resistance is reported. BOTTOM RIGHT: Calibration trials, where (starting from the dummy), filters are added until two consecutive resistances are reported (i.e. two 'Yes' answers for the Yes/No format of the task). If performing a 2IFC task, the required 'Yes' answers for the calibration trials are replaced by correct 2IFC answers. Following this, one final calibration trial is performed with one less filter, to determine the starting value for the task trials. TOP RIGHT: Task trials, where cumulative task accuracy at each trial is transformed (using a beta distribution) into the distribution of underlying accuracies that could have produced the task performance. An upper bound (here 80%) and a lower bound (here 60%) is used to calculate the probability that the participant is performing at the targeted accuracy. If this probability falls below the error risk threshold (here 20%), a filter change is prompted – either the addition of a filter if the accuracy is too low, or the removal of a filter if the accuracy is too high. This continues until either a specified number of trials (here 60 trials) are completed at either one filter number (using the 'constant staircase' task design) or at a range of filter numbers (using the 'roving staircase' task design), the latter requiring no additional trials to be measured that will not be used in the analysis. If a constant staircase is used, the algorithm is stopped at 30 trials, and experimenter intervention can occur every 10 trials subsequently if task performance is drastically altered and no longer deemed acceptable. Figures are adapted from Harrison et al., (2024) under a CCBY license.

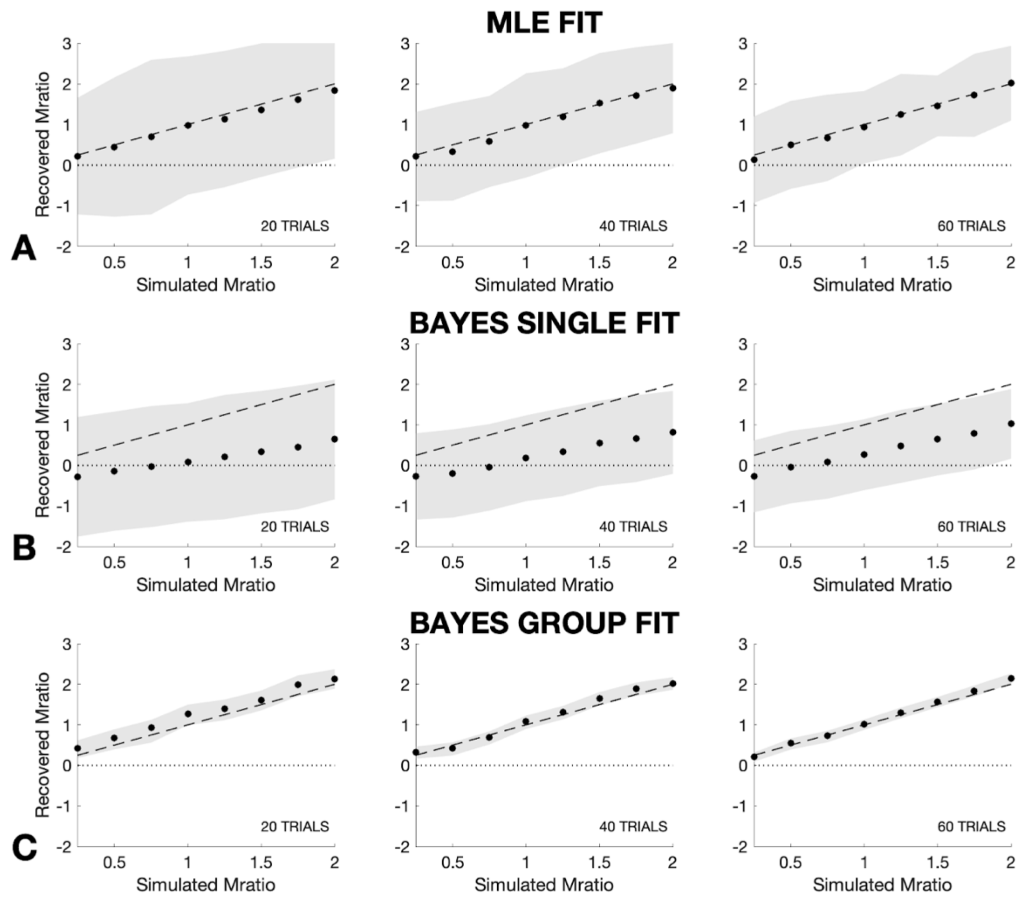

Supplementary Figure 3. Group Mratio recovery for 20, 40 and 60 trials using three different meta-d models. Data were simulated from 8 groups of 60 subjects with group mean Mratio (meta-d' / d') values set to [0.25 0.5 0.75 1.0 1.25 1.5 1.75 2] ± 0.1 (std). All simulated values were generated from data where d' ~ N(1, 0.1) and c ~ N(0, 0.1), and a confidence scale of 10 rating points was used. A) Simulated vs. recovered Mratio values using maximum likelihood estimation (Maniscalco & Lau, 2012), where the shaded grey areas denote the 95% confidence interval of the estimate. B) Simulated vs. recovered Mratio values using a Bayesian single-subject fit (provided in the HMeta-d toolbox (Fleming, 2017)), where the grey areas denote the 95% highest density interval (equivalent to a 95% credible interval) of the sampled estimate. C) Simulated vs. recovered Mratio values using a hierarchical Bayesian group fit (provided in the HMeta-d toolbox (Fleming, 2017)), where the grey areas denote the 95% highest density interval of the sampled estimate. Dashed lines represent ideal recovery, with dotted lines at zero demonstrating the ability of the model fit to significantly recover group estimates (i.e. when confidence or highest density intervals do not include zero). Figure reproduced from Harrison et al. (2021a) under a CCBY licence.

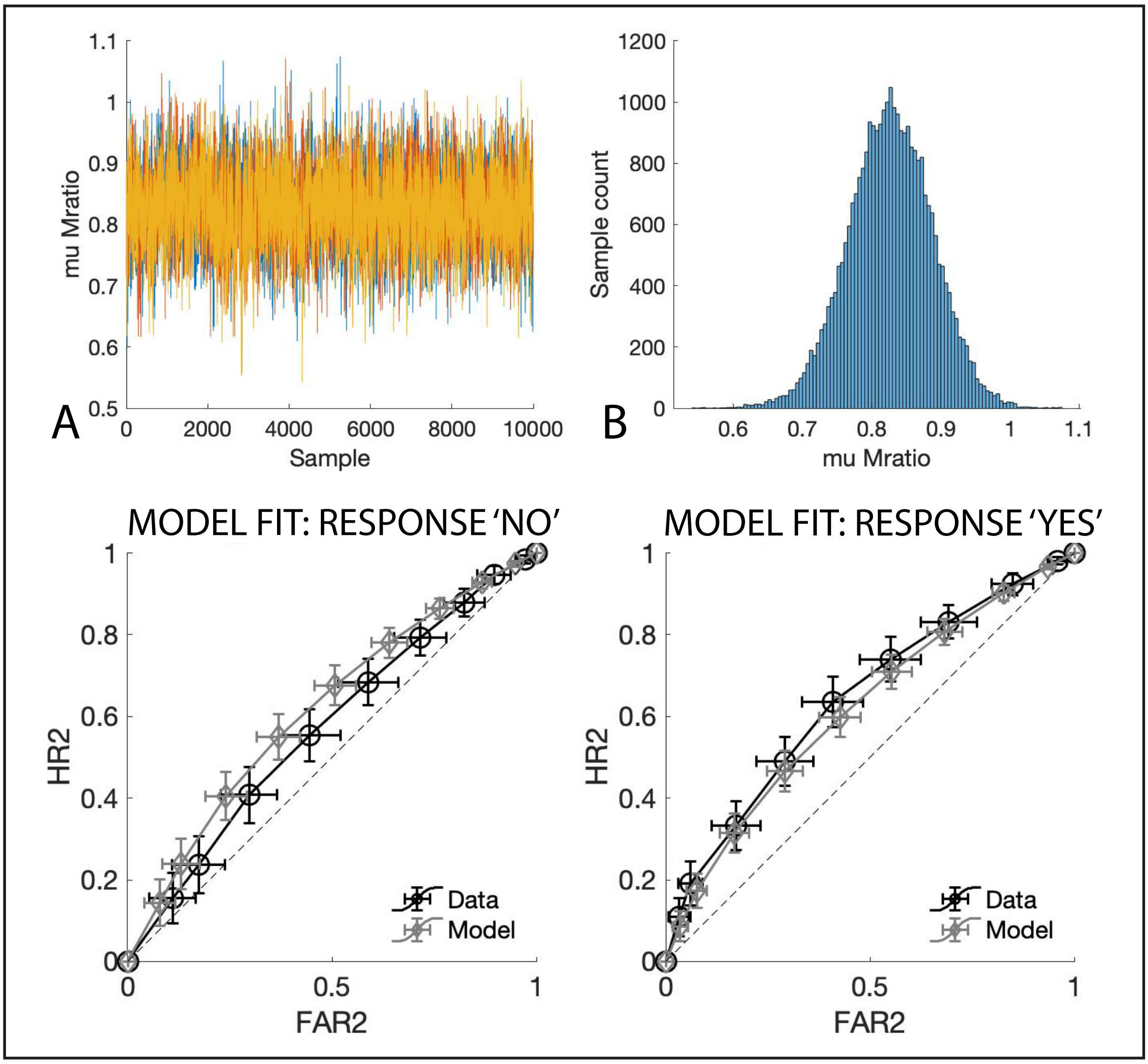

Supplementary Figure 4. Samples from each of three MCMC chains for the group parameter mu Mratio, demonstrating excellent mixing and no evidence of local minima. B) All samples aggregated in a histogram to demonstrate the estimation of the group parameter mu Mratio. C & D) Demonstration of the model fit by comparing the observed and model estimates of the Type 2 ROC curves for both 'No' and 'Yes' responses (regarding the presence of an added inspiratory resistance).

2. Supplementary Anterior Insula Seed Analysis

We performed an additional set of exploratory analyses to investigate the relationship between the rsFC of the anterior insular cortex (aIC) and our interoceptive properties. The aIC ROI was defined using a mask taken from the Brainnetome Atlas of the bilateral ventral and dorsal aIC (Fan et al., 2016). These masks were thresholded at an aIC atlas structure probability of 50% and binarised before being transformed from standard to functional space for each participant (and re-thresholded at a probability of 90% within individual-subject space). These analyses identified significant rsFC differences across metacognitive bias, interoceptive sensitivity and metacognitive insight measures.

Metacognitive Bias

Anterior Insula Average

Metacognitive bias was positively correlated with the rsFC between the bilateral aIC seeds and the lingual gyri and the right occipital fusiform gyrus (Supplementary Figure 5). However, after controlling for GAD-7 scores, no significant correlations remained.

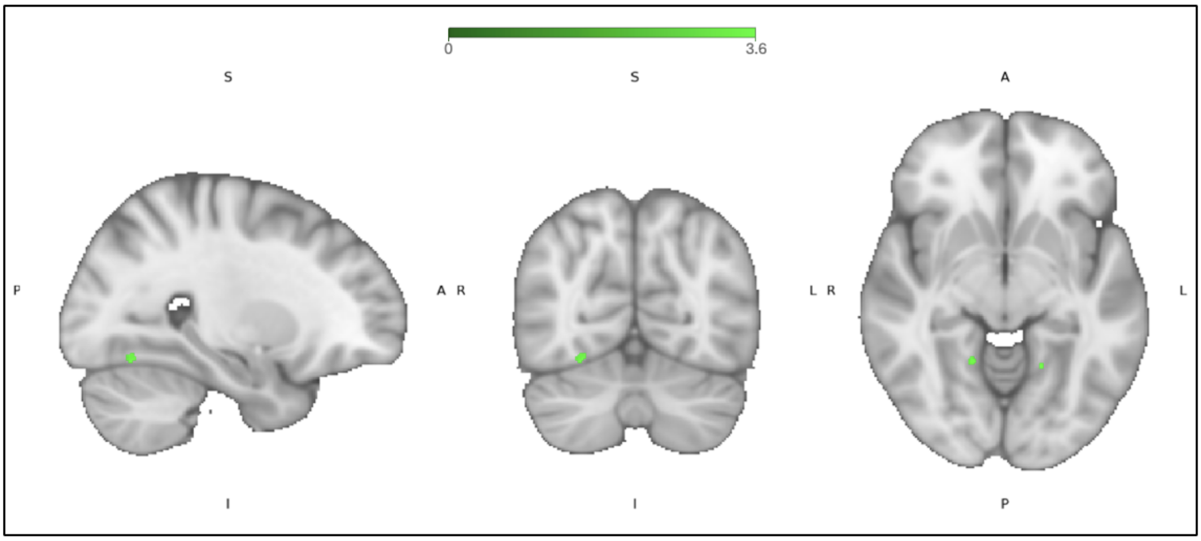

Supplementary Figure 5. Regions demonstrating significant rsFC with the anterior insula correlated with metacognitive bias. The sagittal (left) and coronal plane (middle) exhibit the significant rsFC cluster within the right occipital fusiform gyrus, while the axial plane (right) illustrates the rsFC clusters within the right and left lingual gyri. Statistical maps were derived using permutation testing ($p < 0.05$) and threshold-free cluster enhancement (family-wise error corrected for multiple comparisons). These maps were colour-rendered and superimposed on a MNI standard (1x1x1x1 mm) brain.

Right Anterior Insula > Left Anterior Insula

Metacognitive bias also demonstrated a lateralised connectivity profile, whereby compared to the left aIC seed, greater rsFC between the right aIC seed and the bilateral cingulate gyri (both anterior and posterior divisions), lingual gyri, cerebellar regions, thalami and hippocampi and the left temporal occipital fusiform cortex was positively associated with metacognitive bias (Supplementary Figure 6). Even after controlling for GAD-7 scores, greater rsFC between all the above-mentioned regions and the right aIC seed, when compared to the left aIC seed, remained significantly positively correlated with metacognitive bias (Supplementary Figure 6). Notably, many of these significant connectivity clusters increased in size when GAD-7 scores were included in the GLM.

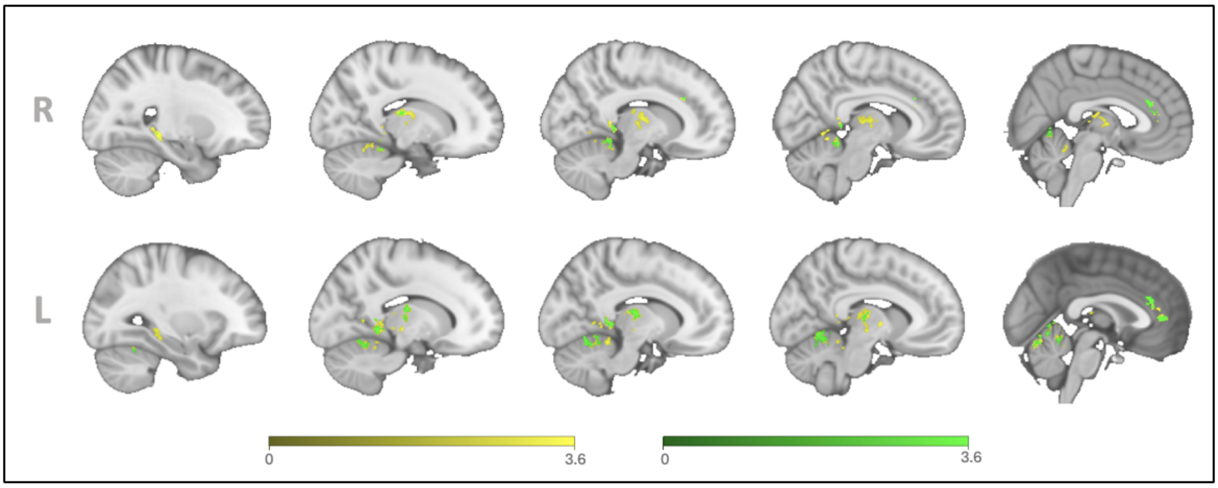

Supplementary Figure 6. Regions exhibiting right > left anterior insula rsFC correlated with metacognitive bias before and after controlling for GAD-7 scores. The green clusters represent the rsFC of the aIC-seeds that were significantly correlated with metacognitive bias before controlling for GAD-7 scores. These have been overlayed by the larger yellow clusters that represent the rsFC of the aIC-seeds which significantly correlated with metacognitive bias after controlling for GAD-7 scores. The 'R' symbol indicates the row of images that depict the right hemisphere, while the 'L' indicates the row of left hemisphere images. Statistical maps were derived using permutation testing ($p < 0.05$) and threshold-free cluster enhancement (family-wise error corrected for multiple comparisons). These maps were colour-rendered and superimposed on a MNI standard (1x1x1x1 mm) brain.

Interoceptive Sensitivity

Left Anterior Insula > Right Anterior Insula

When compared with the right aIC seed, greater rsFC between the left aIC seed and the left central opercular cortex, Heschl's gyrus, planum temporale, IC, planum polare, postcentral gyrus, frontal opercular cortex, superior temporal gyrus (anterior division), inferior frontal gyrus (pars triangularis) and putamen, were positively correlated with interoceptive sensitivity (Supplementary Figure 7). The only connectivity cluster that became insignificant after controlling for GAD-7 scores was located in the inferior frontal gyrus (pars triangularis).

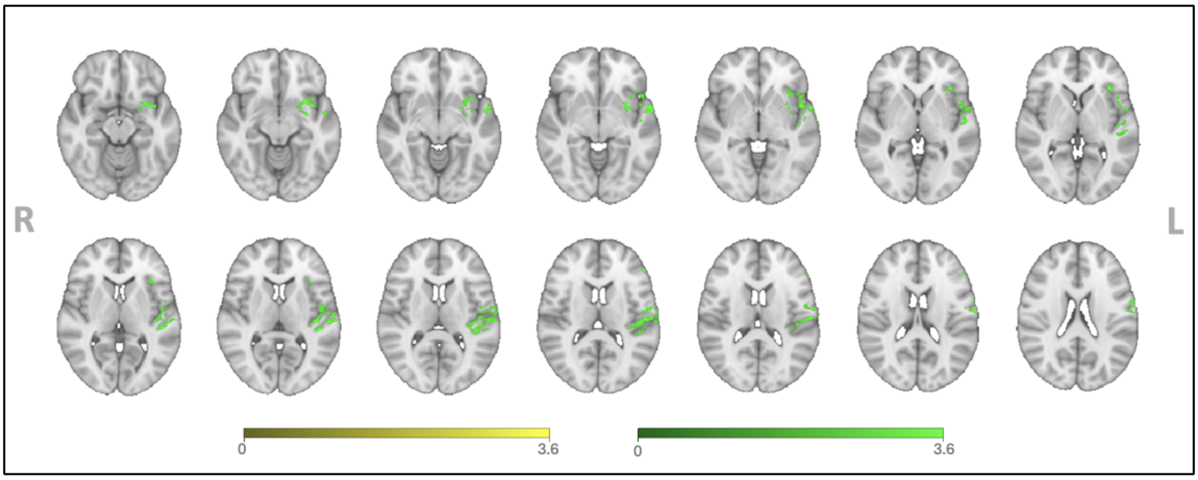

Supplementary Figure 7. Regions exhibiting left > right anterior insula rsFC correlated with interoceptive sensitivity. The left and right brain hemispheres of the images are depicted by the 'R' (right) and 'L' (left) symbols on either side of the figure. Statistical maps were derived using permutation testing (p < 0.05) and threshold-free cluster enhancement (family-wise error corrected for multiple comparisons). These maps were colour-rendered and superimposed on a MNI standard (1x1x1x1 mm) brain.

Metacognitive Insight

Right Anterior Insula > Left Anterior Insula

When comparing the left and right aIC seeds, greater rsFC between the right aIC seed and the bilateral cerebellar nuclei, the left occipital fusiform gyrus, lingual gyrus and temporal occipital fusiform gyrus as well as the right pre- and post-central gyri and the IC were found to be positively correlated with metacognitive insight (Supplementary Figure 8). After controlling for GAD-7 scores, the significant rsFC patterns described above remained largely unchanged other than a very slight increase in the size of significant clusters.

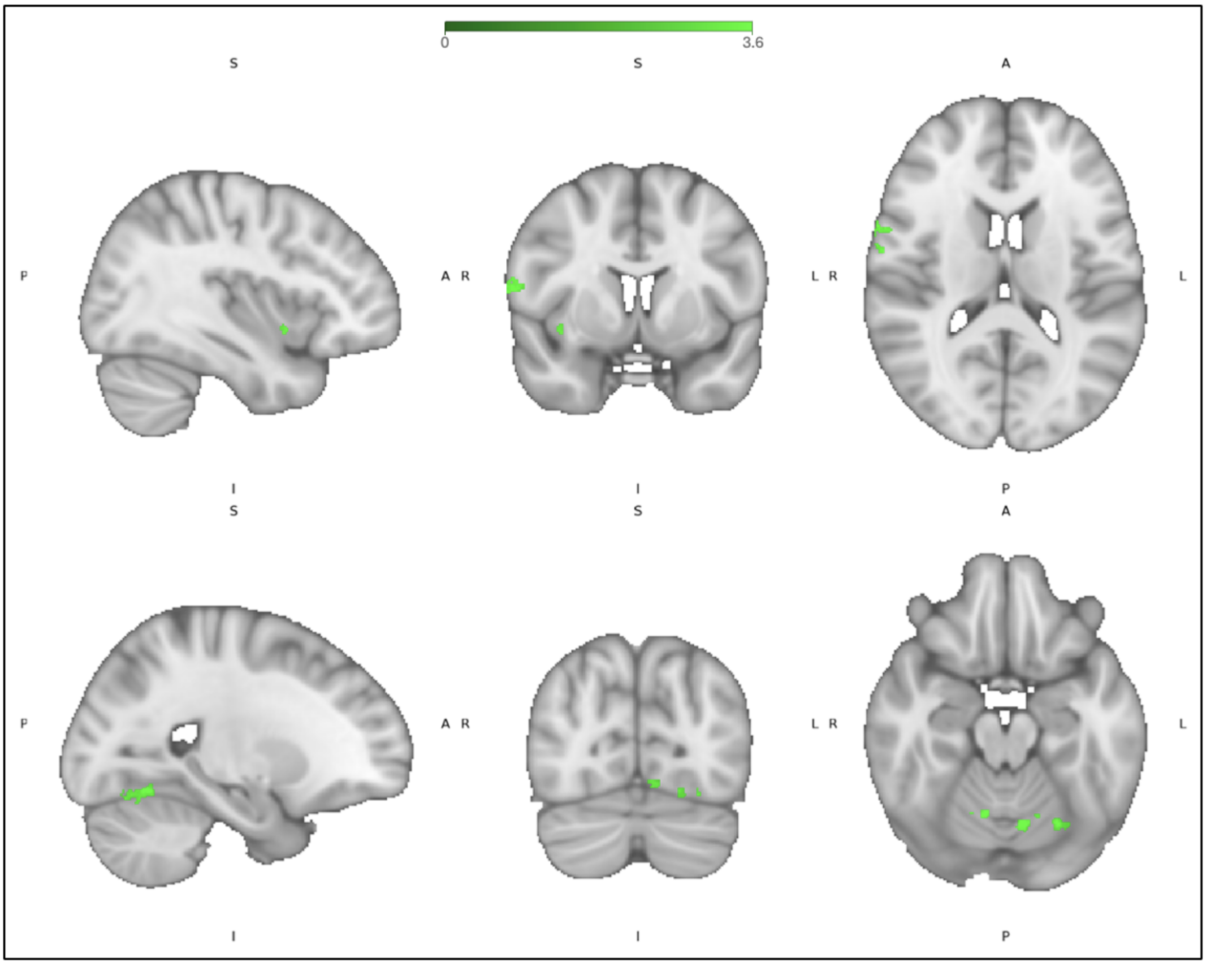

Supplementary Figure 8. Regions exhibiting right > left anterior insula rsFC correlated with metacognitive insight. The top row of images depicts the more anterior significant rsFC clusters (the right pre- and post-central gyri and the right IC). The bottom row highlights the more posterior significant rsFC clusters (the bilateral cerebellar nuclei, left occipital fusiform gyrus, left lingual gyrus and left temporal occipital fusiform gyrus). Statistical maps were derived using permutation testing ($p < 0.05$) and threshold-free cluster enhancement (family-wise error corrected for multiple comparisons). These maps were colour-rendered and superimposed on a MNI standard (1x1x1x1 mm) brain.

3. Pairwise Correlations of Behavioural Variables

Pearson's R Correlation Matrix

	Interoceptive Sensitivity	Decision Bias	Metacognitive Bias	Metacognitive Performance	GAD-7
Interoceptive Sensitivity	1.0000	0.1501	0.0901	0.0665	0.2721
Decision Bias	0.1501	1.0000	0.2791	−0.0439	−0.1219
Metacognitive Bias	0.0900	0.2791	1.0000	0.3468	−0.2570
Metacognitive Performance	0.0665	−0.0439	0.3468	1.0000	−0.1318
GAD-7	0.2721	−0.1219	−0.2570	−0.1318	1.0000

Supplementary Table 1. Matrix depicting the Pearson's R correlation coefficients between each behavioural variable pair. A coefficient of 1.0/-1.0 is indicative of a perfect positive/negative relationship and a coefficient of 0 depicts no relationship.

p-value Matrix

	Interoceptive Sensitivity	Decision Bias	Metacognitive Bias	Metacognitive Performance	GAD-7
Interoceptive Sensitivity	NA	0.2328	0.4756	0.5989	0.0284
Decision Bias	0.2328	NA	0.0243	0.7286	0.3334
Metacognitive Bias	0.4756	0.0243	NA	0.0047	0.0388
Metacognitive Performance	0.5989	0.7286	0.0047	NA	0.2951
GAD-7	0.0284	0.3334	0.0388	0.2951	NA

Supplementary Table 2. Matrix depicting the p-value statistical significance of the correlation between each behavioural variable pair. p-values smaller than 0.05 are taken as statistically significant in this instance.

FDR corrected p-value Matrix

	Interoceptive Sensitivity	Decision Bias	Metacognitive Bias	Metacognitive Performance	GAD-7
Interoceptive Sensitivity	NA	0.4655	0.5944	0.6541	0.0945
Decision Bias	0.4655	NA	0.0945	0.7286	0.4763
Metacognitive Bias	0.5944	0.0945	NA	0.0466	0.0970
Metacognitive Performance	0.6541	0.7286	0.0466	NA	0.4763
GAD-7	0.0945	0.4763	0.0970	0.4763	NA

Supplementary Table 3. Matrix depicting the p-value statistical significance of the correlation between each behavioural variable pair after conducting FDR corrections for multiple comparisons. p-values smaller than 0.05 are taken as statistically significant in this instance.

4. Supplementary Amygdala-Seed, Gender x Decision Bias Interaction

Decision Bias

Left Amygdala

The amygdala-seed resting-state analyses which utilised decision bias as the explanatory variable identified a two-way interaction effect, with significant left-dominant amygdala rsFC differences between males and females. Specifically, in females, increases in decision bias were associated with greater connectivity between the left amygdala seed and the inferior temporal regions (temporal occipital fusiform cortex, inferior temporal gyrus and lingual gyrus; predominantly in the left hemisphere), in comparison to males (Supplementary Figure 9) in a two-way interaction. After controlling for GAD-7 scores, the rsFC patterns described above were no longer significant.

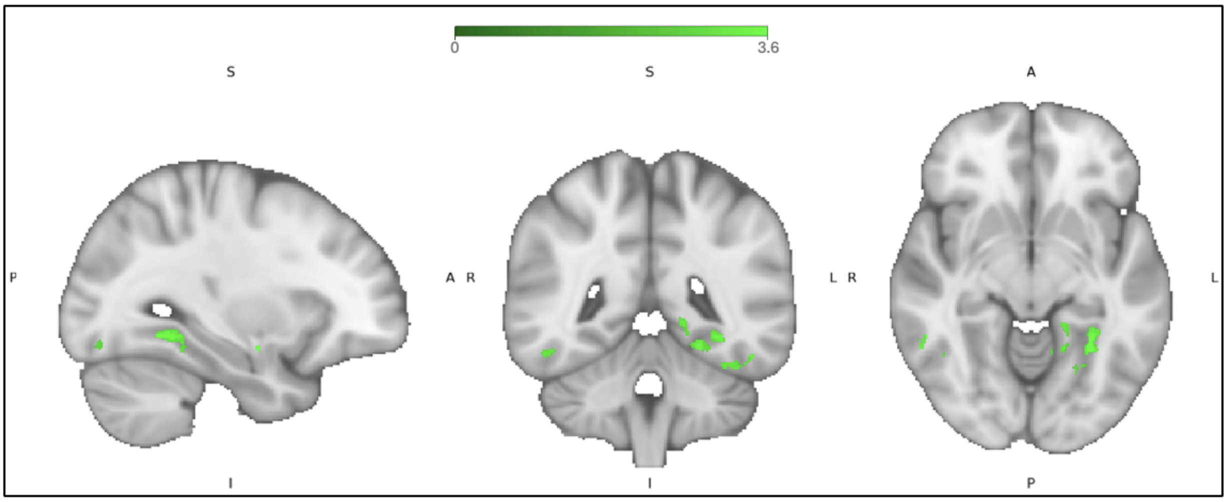

Supplementary Figure 9. Regions demonstrating significant left > right amygdala-seed rsFC in females > males that correlated with decision bias. Statistical maps were derived using permutation testing ($p < 0.05$) and threshold-free cluster enhancement (family-wise error corrected for multiple comparisons). These maps were colour-rendered and superimposed on a MNI standard (1x1x1x1 mm) brain.

5. Similarities Between Amygdala rsFC Patterns with Increases in MAIA and MB Scores.

Metacognitive Bias and MAIA

Our sensitivity analyses revealed that greater rsFC between the right amygdala seed and the insula cortex, corresponded with increased MAIA scores, in a similar way to what was observed between the amygdalae and insula as MB scores increased. Similar patterns were identified when comparing the right amygdala x MAIA contrast with the right amygdala x MB contrast.

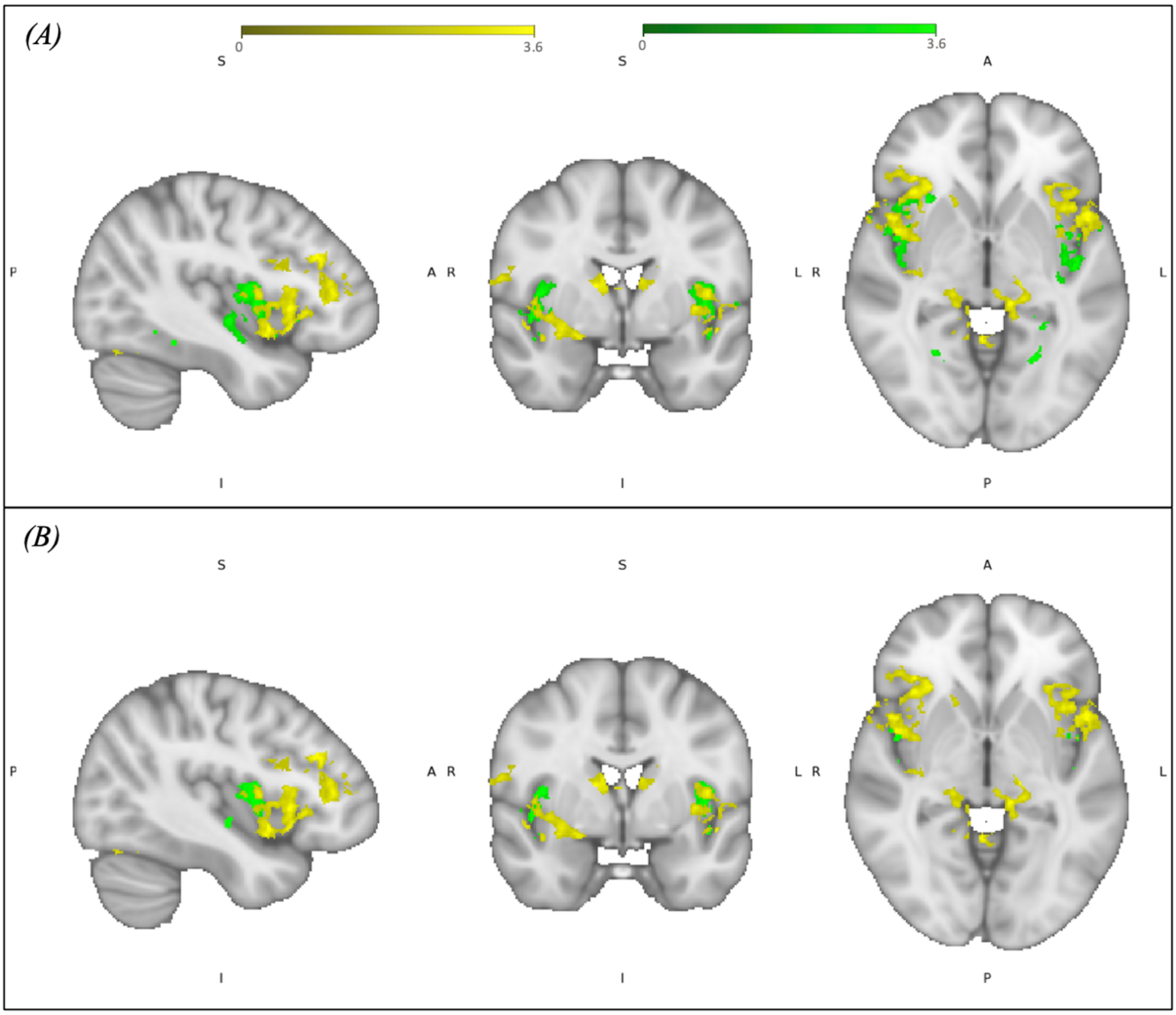

Supplementary Figure 10. Regions with significant rsFC to the right amygdala correlated with MAIA compared with regions with significant amygdalae rsFC correlated with MB. (A) The green clusters depict the Z-scores in regions that showed significant connectivity with the amygdalae on average that correlated with MB, while the yellow clusters (overlayed) illustrate the significant Z-scores in similar regions that showed significant connectivity with the right amygdala seed that correlated with MAIA scores. (B) The green clusters depict the Z-scores in regions that showed significant connectivity with the right amygdala seed that correlated with MB, while the yellow clusters (overlayed) illustrate the significant Z-scores in similar regions that correlated with MAIA scores. Statistical maps were derived using permutation testing (p

$<$ 0.05) and threshold-free cluster enhancement (family-wise error corrected for multiple comparisons). These maps were colour-rendered and superimposed on an MNI standard (1x1x1x1 mm) brain. NOTE: the amygdala average x MAIA contrast did not reveal any significant results.

Supplementary References

Fleming, S. M. (2017). HMeta-d: hierarchical Bayesian estimation of metacognitive efficiency from confidence ratings. *Neuroscience of consciousness*, *2017*(1), nix007. https://www.ncbi.nlm.nih.gov/pmc/articles/PMC5858026/pdf/nix007.pdf

Harrison, O. K., Garfinkel, S. N., Marlow, L., Finnegan, S. L., Marino, S., Köchli, L., Allen, M., Finnemann, J., Keur-Huizinga, L., & Harrison, S. J. (2021a). The Filter Detection Task for measurement of breathing-related interoception and metacognition. *Biological Psychology*, *165*, 108185.

Harrison, O. K., Köchli, L., Marino, S., Marlow, L., Finnegan, S. L., Ainsworth, B., Talks, B. J., Russell, B. R., Harrison, S. J., & Pattinson, K. T. (2024). Gender Differences in the Association Between Anxiety and Interoceptive Insight. *European Journal of Neuroscience*, *61*(1), e16672.

Maniscalco, B., & Lau, H. (2012). A signal detection theoretic approach for estimating metacognitive sensitivity from confidence ratings. *Consciousness and Cognition*, *21*(1), 422-430.
